# Evaluating Bio-Inspired Metaheuristics for Dynamic Surgical Scheduling: A Resilient Three-Stage Flow Shop Model Under Stochastic Emergency Arrivals

**DOI:** 10.3390/biomimetics11030183

**Published:** 2026-03-03

**Authors:** Marcelo Becerra-Rozas, Bady Gana, José Lara, Andres Leiva-Araos, Broderick Crawford, José M. Gómez Pulido, Cristian Contreras, José J. Caro-Miranda, Miguel García-Remesal

**Affiliations:** 1 Escuela de Ingeniería Informática, Pontificia Universidad Católica de Valparaíso, Avenida Brasil 2241, Valparaíso 2362807, Chile; bady.gana@pucv.cl (B.G.); jose.lara.a01@mail.pucv.cl (J.L.); broderick.crawford@pucv.cl (B.C.); 2 Center for Interdisciplinary Research in Biomedicine, Biotechnology and Well-Being (CID3B), Pontificia Universidad Católica de Valparaíso, Av. Brasil 2950, Valparaíso 2362807, Chile; 3 Instituto Data Science, Universidad del Desarrollo, Av. La Plaza 680, Las Condes, Santiago 7610658, Chile; 4 School of Computing, University of North Florida, 1 UNF Dr., Jacksonville, FL 32224, USA; 5 Department of Computing Sciences, Universidad de Alcalá (UAH), Health Computing & Intelligent Systems Research Group, 28805 Alcalá de Henares, Spain; jose.gomez@uah.es; 6 The Ramón y Cajal Health Research Institute (IRYCIS), Madrid, Ctra. de Colmenar Viejo, Km. 9100, 28034 Madrid, Spain; 7 Biomedical Informatics Group, Department of Artificial Intelligence Escuela Técnica Superior de Ingenieros Informáticos, Universidad Politécnica de Madrid, Campus de Montegancedo s/n, 28660 Boadilla del Monte, Spain; cristian.contreras.gomez@alumnos.upm.es (C.C.); mgremesal@fi.upm.es (M.G.-R.); 8 Escuela de Medicina, Sede Patagonia, Universidad San Sebastián, Puerto Montt 5501842, Chile; jose.caro@uss.cl; 9 Unidad de Epidemiología, Hospital Puerto Montt, Puerto Mont 5501842, Chile

**Keywords:** surgical scheduling, population-based metaheuristics, stochastic emergency arrivals, three-stage flow shop, operational resilience

## Abstract

Optimal surgical scheduling necessitates a strategic balance between elective efficiency and responsiveness to stochastic emergency arrivals. This study evaluates a Genetic Algorithm alongside discretized variants of Particle Swarm Optimization, the Secretary Bird Optimization Algorithm, and the Mantis Shrimp Optimization Algorithm. These algorithms are assessed within a dynamic three-stage flexible flow shop model under no-buffer blocking constraints. Findings from 300 Monte Carlo replications demonstrate that while the Genetic Algorithm achieves peak global efficiency, discretized bio-inspired algorithms reach a comparable statistical efficiency frontier. Notably, the discretized Secretary Bird Optimization Algorithm facilitates superior emergency integration by maintaining natural capacity buffers, whereas the aggressive local optimization characteristic of alternative methods often triggers resource saturation in recovery units. These results indicate a potential recovery of 90 annual operating hours per theater.These results indicate a potential recovery of 90 annual operating hours per theater, representing a 6.7% increase in resource utilization efficiency. This improvement provides a critical data-driven capacity margin to mitigate the non-prioritized (Non-GES) surgical backlog in Chilean public hospitals.

## 1. Introduction

The optimization of surgical scheduling (SS) is a fundamental pillar of modern hospital management, requiring the efficient allocation of finite resources (operating rooms, medical personnel, and specialized equipment) across a defined planning horizon. Effective SS strategies maximize the utilization of these scarce assets while minimizing patient waiting times, thereby enhancing clinical care quality. However, the problem remains inherently complex due to a convergence of operational constraints, including restricted theater and staff availability, inherent variability in surgical durations, and the limited capacity of postoperative recovery beds. Furthermore, the system must remain resilient to unforeseen emergency procedures. Given the NP-hard combinatorial nature of SS and the necessity of satisfying multiple simultaneous constraints, research has pivoted from exact mathematical models toward advanced heuristic and metaheuristic approaches.

Structurally, the SS problem resembles the classical Job Shop Scheduling Problem (JSSP) or its flexible flow variants, where a set of “jobs” (i.e., surgeries) must be processed across multiple “stations” or “machines” (such as anesthesia units, operating rooms, and recovery areas) in a specific sequence, while adhering to resource-sharing constraints. This analogy allows the modeling of surgical planning as a well-established combinatorial optimization problem, where feasible solutions must satisfy precedence relations, availability windows, and compatibility constraints among physical and human resources.

In this context, Genetic Algorithms (GA) and Particle Swarm Optimization (PSO) have been successfully applied to various scheduling problems [1,2], aiming to minimize performance indicators such as the makespan, balanced resource utilization, or total patient waiting time.

Within this framework, Bai et al. [3] proposed a SS model for ambulatory procedures in a large public hospital in China, formulating it as a three-stage sequencing problem (a case of flexible flow shop scheduling) and applying a GA to minimize the makespan of the surgical plan. Their approach, validated with real-world data, demonstrated significant improvements in resource utilization and reductions in patient waiting times, while also allowing for the integration of emergent procedures. Building upon this framework, the present study executes a computational replication of the original experiment using simulated data derived from the clinical, operational, and resource-related characteristics reported in [3]. The experimental design involves the implementation of a genetic algorithm similar to that in the original study, along with discrete versions of population-based metaheuristic algorithms: PSO, Secretary Bird Optimization (SBOA), and Mantis Shrimp Optimization Algorithm (MShOA), with the objective of comparing their performance in a controlled setting. The comparison focuses on classical metrics such as solution quality (in terms of makespan), result stability, and convergence speed, and incorporates statistical tests to assess significant differences. Beyond replicating an existing model, this study represents a preliminary stage of a broader research initiative aimed at evaluating the potential of population-based metaheuristics in real-world SS scenarios. The ultimate goal is to establish a robust methodological foundation that enables the future adaptation and deployment of these techniques in actual hospital settings in Chile, thereby contributing to the development of more efficient and context-sensitive planning solutions.

The main contributions of this work are as follows:

- The adaptation and comparative evaluation of four population-based metaheuristics, GA, dPSO, SBOA, and dMShOA, on a SS model structured as a resilient three-stage flow shop consisting of Anesthesia Preparation Room (APR), Operating Room (OR), and Post-Anesthesia Recovery Room (ARR) stages.
- The design of a reproducible experimental framework using Monte Carlo simulation to analyze algorithmic behavior under strict no-buffer blocking constraints and stochastic emergency arrivals.
- The generation of evidence identifying a performance hierarchy where modern bio-inspired metaheuristics reach the statistical efficiency frontier of established genetic algorithms.
- The establishment of a methodological baseline to support the upcoming transition from simulated environments to empirical calibration using real-world data from the Los Lagos Region of Chile.

The document is organized as follows. Section 2 reviews related work regarding optimization models and the Chilean healthcare context. Section 3 defines the mathematical model and the Job Shop approach. Section 4 details the proposed metaheuristic approaches and discretization mechanisms. Section 5 describes the experimental setup and benchmark instances. Section 6 provides an in-depth analysis of the experimental results and algorithmic performance. Finally, Section 7 presents the conclusions and directions for future research.

## 2. Related Work

### 2.1. Optimization Models and Metaheuristic Approaches in Surgical Scheduling

The SS procedures is widely recognized as one of the most resource-intensive and operationally demanding processes in healthcare management. Operating room activities account for approximately 40% of total hospital expenditures, encompassing personnel, equipment, and facility costs [4]. To address this complexity, the literature categorizes surgical planning into three hierarchical decision levels: strategic, tactical, and operational. However, persistent coordination gaps across these layers often impede the effective integration of analytical advancements into clinical practice [5,6,7]. This misalignment adversely affects critical performance indicators, such as last-minute cancellations and patient satisfaction, thereby underscoring the necessity for integrated, context-sensitive scheduling frameworks that bridge the gap between theoretical optimization and clinical reality.

Initial modeling efforts in SS were predominantly deterministic, assuming fixed procedure durations and uninterrupted resource availability. Notable early contributions include the works of Valisiar et al. [8] and Fei et al. [9] the latter still regarded as a foundational reference despite its publication in 2010. As empirical evidence began to highlight the substantial variability inherent in real-world surgical operations, researchers shifted toward stochastic models capable of capturing uncertainty. Among these, the two-stage stochastic formulation by Denton et al. [10] stands out for its methodological rigor. In parallel, robust optimization approaches were developed to enhance the stability of solutions under varying operational scenarios [11,12], while fuzzy modeling techniques emerged as an effective means of incorporating imprecise or ambiguous clinical parameters through fuzzy numbers [13].

Furthermore, recent studies have emphasized that addressing high variability in emergency admissions requires advanced forecasting frameworks. The application of recurrent neural networks, specifically Long Short-Term Memory models incorporating contextual variables, has demonstrated significant potential in improving the predictability of emergency arrivals for long-term resource allocation [14]. Complementary efforts have explored the use of regressor algorithms and digital trend analysis to identify representative search terms, enabling hospital administrations to anticipate unforeseen surges in emergency care demand [15].

Most studies model SS as a Job Shop or Flexible Flow Shop problem, typically following the sequence *APR–OR–ARR*, which underscores the NP-hard nature of the problem [3]. To tackle this complexity, metaheuristic approaches have become essential tools. GA have demonstrated a well-established trajectory, with applications ranging from master planning to daily sequencing tasks [16,17]. PSO, in contrast, has received comparatively less attention; however, results reported in flow shop contexts suggest that it offers competitive performance [18]. This underrepresentation of PSO in the SS literature provides motivation for the GA–PSO comparison conducted in the present study.

Alternative strategies, including Simulated Annealing, Tabu Search, Variable Neighborhood Search, and Ant Colony Optimization, alongside hybrid discrete-event simulation frameworks, have expanded the methodological capabilities for modeling uncertainty and evaluating dispatching rules [19]. Nevertheless, the simultaneous integration of the operating room, Post-Anesthesia Care Unit (PACU), Intensive Care Unit (ICU), and personnel constraints remains a significant challenge, particularly in resource-constrained hospital settings [12,20].

### 2.2. Status Quo of Surgical Scheduling in Chile

In Chile, SS remains predominantly manual in both public hospitals and private clinics. Planning is typically organized through fixed surgical tables per specialty, with limited adaptability to real demand and without integration of predictive or optimization systems. In the public sector, most operating rooms are scheduled using spreadsheets or basic administrative tools without algorithmic logic. This often leads to suboptimal utilization of surgical time and resources, frequently reported at around 60% of total available time [21,22].

Major bottlenecks include high elective-surgery suspension rates (commonly around 10–12%), limited availability of PACU/ICU beds, inflexibility in the face of emergencies, and weak coordination between clinical and administrative units [23]. Additionally, structural issues include the relative shortage of anesthesiologists (particularly pediatric subspecialists) [24], inaccurate estimation of surgical durations [25,26], and low interoperability across outpatient scheduling, Electronic Health Record, logistics, and supplies [27]. These factors exacerbate workflow fragmentation.

From a policy standpoint, national targets include keeping elective-surgery suspensions below 7%, and ministerial communications have aimed for operating room utilization levels near 80%; however, the latter is not codified as a COMGES indicator [25]. The implementation of preoperative units has also been promoted to reduce preventable errors and strengthen patient readiness [23]. Despite these initiatives, diagnostic assessments repeatedly show a gap between planning and execution, driven by complex change management, rigid organizational structures, limited clinical governance, and the absence of analytics tools for operational decision-making [21,22].

In this context, incorporating engineering and data-science techniques such as genetic algorithms, stochastic optimization, and population-based heuristics offers a promising avenue to improve scheduling efficiency [28]. National academic evidence in simulated settings reports reductions in idle time, higher surgical throughput, and fewer assignment errors. For instance, Latorre-Núñez et al. in [29] show that an optimization-based operating room scheduling model increased scheduled surgeries by up to 30% and improved theatre utilization by nearly 20% in simulation, suggesting a viable pathway to bridge the gap between installed capacity and actual production.

## 3. Methodology

### 3.1. Problem Definition and Mathematical Model

Conceptually, the proposed model treats the surgical flow as a synchronized journey through three critical hospital stages: APR, the OR and ARR. The model’s logic is specifically designed to manage the interplay between stable elective demand and unpredictable emergency arrivals. In a real-world scenario, the system must prevent “cascading delays”; for instance, if the ARR is saturated, a patient cannot leave the OR, effectively locking that theater and forcing the cancellation of subsequent elective surgeries. To mitigate this, the model enforces no-buffer blocking constraints, ensuring that resource allocation is optimized under these strict clinical dependencies. This approach aims to enhance operational resilience and provide a data-driven decision support tool for effective surgical waitlist management.

The SS problem is formalized as a combinatorial optimization task involving scarce and interdependent resources. This section defines the scheduling framework, introduces the notation, and presents the Mixed-Integer Programming (MIP) formulation designed to minimize makespan while ensuring operational resilience.

#### 3.1.1. A Job Shop Approach to Surgical Case Scheduling

Consistent with the perioperative literature and inspired by the three-stage framework proposed by Bai et al. [3], we model the surgical flow as a Flexible Job Shop Scheduling Problem (FJSSP). However, our approach introduces significant modifications to the original framework to better capture the stochastic dynamics of modern hospitals. Unlike the baseline model, we explicitly integrate a non-preemptive rescheduling control loop and a multi-component objective function that prioritizes clinical safety windows for emergency cases.

This sequence is enforced by the clinical necessity of the procedures, treating each surgery *J* is treated as a job consisting of o=3 operations that must be processed on specific resources selected from parallel pools at each stage. A critical feature of this model is the enforcement of a no-buffer blocking constraint: a patient cannot vacate a resource at stage *k* (e.g., the OR) until a resource at stage k+1 (e.g., an ARR bed) is available and setup. This reflects the operational reality where downstream saturation propagates congestion upstream, effectively locking the system.

#### 3.1.2. Notation

The system is formalized as a MIP model. Table 1 presents the sets, parameters, and decision variables used in the formulation.

#### 3.1.3. Mathematical Formulation

The objective function (Equation (1)) formalizes a strategic trade-off between global throughput and clinical responsiveness. It is composed of three primary terms:

(1) $$\mathrm{Minimize}\;x_{\tau }+\alpha \sum_{j\in J}\sum_{i\in I}\sum_{r\in R}x_{j_{i}}^{r}\cdot z_{j_{i}}^{r}+\omega \sum_{E\in E}\sum_{i\in I}\sum_{r\in R}x_{E_{i}}^{r}\cdot z_{E_{i}}^{r}$$

This formulation integrates three primary components: the makespan ($x_{\tau }$), which minimizes the total completion time to maximize productivity, and weighted penalty terms for elective and emergency start times. The elective term, weighted by α, promotes early starts to mitigate inefficient gaps in the schedule. Crucially, the dominance condition ω≫α is dictated by clinical triage protocols rather than arbitrary parameter tuning. From a clinical governance perspective, delaying an emergency intervention carries a substantial risk of physiological deterioration (including mortality or permanent disability) whereas elective delays predominantly result in administrative costs. Consequently, this hierarchy ensures that the metaheuristic prioritize patient safety over marginal gains in elective efficiency.

As demonstrated in our results (Section 6.4), this weighting strategy is empirically validated by its ability to consistently maintain emergency integration delays below the critical 60-min safety threshold across all stochastic scenarios.

The model is subject to the following operational and safety constraints:

- Clinical sequence: Each surgery must complete one stage before starting the next.
  (2) $$L_{j_{i}}^{u}-t_{j_{dp}}^{u}+H\left(1-\theta_{ru}^{j}\right)\geq L_{j_{k}}^{r},\;\forall j\in J\cup E$$
- Personnel exclusivity: Staff members can only participate in one surgery at a time.
  (3) $$x_{Q_{dp}}^{r}\geq x_{j_{dp}}^{r}+t_{i_{dp}}^{r}-H\left(1-y_{r}^{jQ}\right)$$
- Resource sequence and cleaning: Includes the required sanitation period between procedures.
  (4) $$x_{Q_{i}}^{r}\geq \left(x_{j_{i}}^{r}+t_{i_{dp}}^{r}+t_{i}^{c}\right)-H\left(3-y_{r}^{jQ}-z_{j_{i}}^{r}-z_{Q_{i}}^{r}\right)$$
- Blocking and stay limits: Enforces the no-buffer policy where upstream resources remain blocked until downstream capacity is available.
  (5) $$L_{j_{i}}^{r}-x_{j_{i}}^{r}-t_{i_{dp}}^{r}\cdot z_{j_{i}}^{r}\leq b_{i}$$
  The enforcement of the no-buffer blocking constraint is a clinical necessity rather than a mere mathematical abstraction. In a surgical flow, “jobs” are human patients whose physiological transitions (especially between the OR and ARR) require continuous specialized monitoring. An intermediate buffer (common in industrial flow shops) would imply leaving a post-operative patient in an unsupervised area. Thus, if an ARR bed is unavailable, the OR must function as a temporary recovery station, effectively “locking” the upstream resource to ensure patient safety, a phenomenon known as “operating room holding” [5].
- Makespan boundary: Ensures the total horizon covers all operations and cleanups.
  (6) $$x_{\tau }-L_{j_{i}}^{r}\geq t_{i}^{c},\;\forall i\in I_{last}$$

Emergency Integration Constraints:

- Arrival synchronization: An emergency cannot be scheduled before it physically arrives at the facility.
  (7) $$x_{E_{1}}^{r}\geq t_{E}^{arr}\cdot z_{E_{1}}^{r},\;\forall E\in E,r\in R_{1}$$
- Admissible delay: Ensures the surgery begins within a clinical safety window to minimize risk.
  (8) $$x_{E_{1}}^{r}\leq \left(t_{E}^{arr}+b_{1}\right)\cdot z_{E_{1}}^{r},\;\forall E\in E,r\in R_{1}$$
- Dynamic resource availability: Pending tasks must respect the updated availability times $e^{r}$ generated by the arrival of an emergency.
  (9) $$x_{Q_{i}}^{r}\geq e^{r}\cdot z_{Q_{i}}^{r},\;\forall Q\in J^{RS}$$
- Non-negativity of temporal variables.
  (10) $$x_{j_{i}}^{r}\geq 0,\;L_{j_{i}}^{r}\geq 0,\;x_{\tau }\geq 0$$

#### 3.1.4. Practical Interpretation of Algebraic Constraints into Hospital Protocols

To ensure that the mathematical model serves as a practical decision-support tool, the key operational constraints are translated into the language of hospital management. Table 2 summarizes the transformation of these algebraic forms into clinical protocols.

The precedence and exclusivity constraints (Equations (2) and (3)) ensure a linear and safe progression of the patient, while the sanitation requirement (Equation (4)) guarantees that hygiene standards are never compromised for the sake of efficiency. Equation (5) is particularly critical for clinical governance, as it models the “hydraulic lock” where OR throughput is paralyzed by downstream recovery saturation. Finally, Equations (7)–(9) manage the integration of stochastic emergencies, ensuring that triage windows are respected without collapsing the elective schedule.

### 3.2. Modeling Uncertainty and Dynamic Rescheduling Policy

We evaluate the operational impact of unexpected arrivals and algorithmic robustness through two simulation environments based on identical discrete-event constraints. The *Elective Mode* ($S_{\mathrm{elective}}$) addresses the planned workload subject to processing-time uncertainty. In this scenario, the schedule remains static throughout the Monte Carlo replication, meaning performance relies solely on the quality of the initial optimization.

The *Emergency Mode* ($S_{\mathrm{emergency}}$), on the other hand, introduces a control loop driven by stochastic urgent arrivals that trigger sequential rescheduling. When an emergency occurs at time $t^{arr}$, the system applies a non-preemptive policy: any job *j* already in execution (where the recorded start time satisfies $Start<t^{arr}$) continues undisturbed. We collect all pending jobs (including previously queued emergencies) along with the new arrival and submit them for re-optimization, prioritizing the incoming emergency.

The “freeze-and-merge” policy reflects the non-preemptive nature of surgical interventions. Unlike manufacturing tasks, a surgery cannot be interrupted once an incision is made without violating the principle of non-maleficence. By “freezing” started procedures and “merging” the new emergency arrival into the pending elective queue, the model respects the ethical and physical reality of the clinical responsibility, ensuring continuity of care for patients already under anesthesia.

Finally, the new partial plan merges with the frozen segment by adjusting task timing after $t^{arr}$. This step enforces post-arrival room availability and intra-job precedence, maintaining consistency with ongoing commitments. This setup allows us to distinguish between the algorithm’s static optimization capability and its adaptability when facing high-priority disruptions.

## 4. Proposed Metaheuristic Approaches

We evaluate four population-based metaheuristics implemented for the three-stage SS setting: GA, dPSO, SBOA, and dMShOA. Each method searches over an identical schedule encoding and is scored by a unified discrete-event simulation routine. This common evaluator constructs the resource-feasible plan under precedence, shared-capacity, and blocking constraints, returning a single composite fitness value; this standardizes feasibility handling and objective accounting, ensuring performance differences reflect search dynamics rather than inconsistencies in decoding or metric computation.

### 4.1. Solution Encoding and Schedule Evaluation

A candidate schedule is encoded as two coupled decision layers: (i) a base priority sequence, denoted by *job_sequence_base*, and (ii) a room-selection map, denoted by *room_assignment*, which specifies for each job and each operation index o∈{1,2,3} the concrete room used at that stage. The decoder evaluates this encoding through a single discrete-event simulator that maintains release-time calendars for all rooms and all staff members, together with per-job timestamps for operation start, processing completion, and resource release, thereby enforcing precedence, shared-capacity constraints, and downstream blocking in one consistent mechanism. Dispatching decisions are generated from a heap-based event queue keyed by an estimated earliest start time and a deterministic job ranking induced by the priority sequence; a numeric counter is included in the event key to guarantee a stable total order even when the job identifiers combine elective integers and emergency string IDs. The simulator also biases dispatching toward flow continuity by giving the next stage of the same job a higher scheduling priority, which reduces unnecessary inter-stage idling when feasible.

Personnel assignment is not part of the encoding and is instead resolved online at dispatch time by selecting, from the operation-specific qualified pool, the staff member with earliest availability, with deterministic tie-breaking by identifier. Each dispatched operation incorporates setup and cleanup times indexed by the job type, while allowing controlled overlap of setup with the predecessor’s tail: setup starts as soon as both the assigned room and the selected staff member are available, but it is also anchored to the predecessor completion through the implemented offset so that processing begins exactly after setup. Precedence is enforced by construction through the per-job predecessor completion times; capacity coupling follows from updating the room and personnel release times at operation completion, and the no-buffer policy is explicitly enforced by delaying the release of the upstream room and personnel from stage “o−1” until the downstream setup begins at stage “*o*”, which propagates congestion upstream when downstream capacity is saturated. Feasibility is screened against operation-specific maximum waiting limits, and the evaluator returns a composite objective that preserves makespan as the dominant term while adding weighted penalties for aggregate start times, total and maximum inter-operation waiting, and room-usage imbalance.

### 4.2. Discretization Mechanisms

The four metaheuristics operate over the same evaluated representation, yet they reach it through different state spaces and update operators. The transition from continuous dynamics to discrete decision spaces constitutes a fundamental architectural component in combinatorial optimization. As highlighted in systematic reviews of the field [30], the selection of a discretization strategy is pivotal for maintaining the search capabilities of the underlying metaheuristic and is not merely a trivial preprocessing step. While the GA is purely discrete, utilizing sequence operators such as order-based crossover and swap mutation, the dPSO, SBOA, and dMShOA algorithms rely on continuous state components. Consequently, these methods require explicit mapping strategies to translate real-valued coordinates into the discrete permutation and room indices required by the simulator.

#### 4.2.1. Sequence Discretization

**Figure 1:**
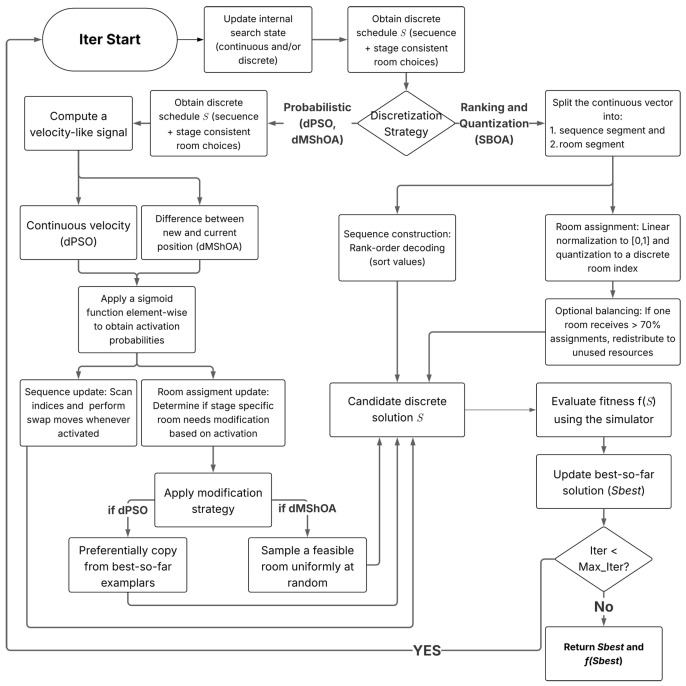
Algorithmic flowchart detailing the discretization mechanisms and state-update procedures for mapping continuous search spaces into discrete scheduling sequences.

For the continuous algorithms, the job sequence is derived using distinct topological mappings, as illustrated in the decision branches of Figure 1. SBOA utilizes rank-order decoding: the first |J| components of the continuous position vector are sorted in ascending order, and the resulting index permutation defines the surgery sequence. dPSO and dMShOA, conversely, maintain an incumbent discrete sequence and apply probabilistic perturbations. In dPSO, the sequence velocity is converted elementwise into swap probabilities via a sigmoid function. This approach follows principled binarization logic where transfer functions and operator transformations are employed to map continuous trajectories into discrete transition probabilities, thereby preserving the delicate balance between exploration and exploitation [31]. The algorithm scans shuffled indices and performs a swap between position *k* and a random target *l* whenever a uniform random draw falls below the computed probability. dMShOA applies an analogous mechanism driven by a velocity-like displacement, effectively modulating the perturbation intensity based on the magnitude of the continuous movement.

#### 4.2.2. Room Assignment Discretization

Room assignment is discretized stage-consistently, as detailed in the corresponding branches of Figure 1, to ensure decisions for o∈{1,2,3} remain within admissible pools. SBOA implements a direct mapping where the relevant segment of the position vector is linearly normalized from the search interval to [0,1], then scaled to an integer index selecting a room from the stage-specific list. To mitigate the risk of convergence to a single room, a post-decoding balancing routine is triggered if any room absorbs more than 70% of assignments while others remain empty, redistributing a subset of jobs to unused resources. dPSO updates the room map through probabilistic edits driven by the sigmoid of a flattened room-velocity vector; activated changes preferentially copy assignments from personal-best or global-best exemplars, falling back to random feasible rooms only when no exemplar is selected. dMShOA employs a simplified stochastic update: the sigmoid of the velocity-like displacement determines whether a room decision is modified, and every activated modification overwrites the current room with a uniformly sampled feasible room from the appropriate stage pool, yielding diversity without reliance on exemplar memory.

### 4.3. Algorithm Overviews

To facilitate the formal description of the metaheuristics, the additional notation used in the operator-level pseudocodes is presented in Table 3.

#### 4.3.1. Genetic Algorithm

The implemented GA follows the canonical evolutionary framework adapted for sequencing problems [32]. The population consists of candidate schedules where individuals are initialized with a randomized priority list and a room assignment designed to promote a balanced initial use of resources. Selection is performed using an inverted roulette-wheel scheme suited for minimization. Recombination combines order-based crossover (OX) on the priority list with single-point exchange for room assignments. Mutation applies swap operations on the priority list and random room perturbations to maintain diversity. The algorithmic steps for the GA operators are formalized in Algorithm 1.
**Algorithm 1** GA operators: selection, crossover, and mutation mechanisms (used inside Algorithm 5).1: **Individual:** S=(π,ρ)2: **Selection:** inverted roulette-wheel3: **Elitism:** copy best $E_{elite}$ to $P_{\mathrm{new}}$4: **while** $|P_{\mathrm{new}}|<N$ **do**5:        Select parents $S_{a}=\left(\pi_{a},\rho_{a}\right)$ and $S_{b}=\left(\pi_{b},\rho_{b}\right)$6:        **if** $RAND\left(\right)<p_{c}$ **then**7:               Apply OX on π and one-point crossover on ρ8:        **else**9:               Offspring ← copies of parents10:        **end if**11:        **if** $RAND\left(\right)<p_{m}$ **then**12:               Swap-mutate π13:        **end if**14:        **if** $RAND\left(\right)<p_{m}$ **then**15:               Perturb ρ16:        **end if**17:        Repair ρ (stage feasibility)18:        Insert offspring into $P_{\mathrm{new}}$19: **end while**

#### 4.3.2. Discrete Particle Swarm Optimization

The dPSO variant adapts the continuous flocking behavior to combinatorial spaces [33]. Each particle stores a discrete position (priority list and room assignment) and continuous velocities for both components. Velocities are updated using standard inertia and attraction toward personal and global bests. A sigmoid mapping converts these velocities into probabilities that trigger discrete moves: swaps in the priority list and edits in room assignments, as shown in Algorithm 2.
**Algorithm 2** dPSO update operators and memory management logic (used inside Algorithm 5).1: **State:** S=(π,ρ), velocities $\left(V^{\pi },V^{\rho }\right)$2: **Memory:** $S^{pbest}$, $S_{\mathrm{best}}$3: Update $V^{\pi },V^{\rho }$ (inertia + attraction to $S^{pbest}$ and $S_{\mathrm{best}}$)4: $p^{\pi }\leftarrow \sigma \left(V^{\pi }\right)$, $p^{\rho }\leftarrow \sigma \left(V^{\rho }\right)$5: Apply swaps in π activated by $p^{\pi }$6: Apply edits in ρ activated by $p^{\rho }$ (copy from $S^{pbest}$ or $S_{\mathrm{best}}$, else random feasible room)7: Set S←(π,ρ)8: Update $S^{pbest}$ if improved (after evaluation)

#### 4.3.3. Secretary Bird Optimization Algorithm

The SBOA is a recent bio-inspired metaheuristic that mimics the hunting strategies of the secretary bird [34]. It operates in a continuous search space, alternating between exploration and exploitation phases based on hunting intervals. Each continuous position vector is decoded into (i) a surgery priority list via rank-order decoding and (ii) room assignments via normalized indexing into the feasible room lists. A balancing routine is applied to reduce extreme room concentration before fitness evaluation, following the procedure in Algorithm 3.
**Algorithm 3** SBOA search phase and rank-order decoding procedure (used inside Algorithm 5).1: **State:** $X\in {\left[L,U\right]}^{d}$2: Choose phase (exploration / exploitation)3: Update *X* (SBOA rule for the chosen phase)4: Decode S=(π,ρ):5:      π← rank-order of the sequence components of *X*6:      ρ← normalized indexing of room components into feasible stage-wise room lists7: Apply room-balancing if assignments are highly concentrated8: Return *S*

#### 4.3.4. Discrete Mantis Shrimp Optimization Algorithm

The dMShOA is based on the foraging and combat behavior of mantis shrimps [35]. The algorithm maintains a continuous position per agent and generates a velocity-like displacement based on foraging, attack, and defense mechanisms. This displacement is transformed into probabilities that trigger swaps and random room reassignments, as detailed in Algorithm 4. The parameter *k* controls the magnitude of certain movements, balancing the exploration–exploitation trade-off.
**Algorithm 4** dMShOA movements and probabilistic discretization logic (used inside Algorithm 5).1: **State:** $X\in {\left[L,U\right]}^{d}$, S=(π,ρ), parameter *k*2: Update *X* using dMShOA movements and compute displacement Δ3: p←σ(Δ)4: Apply swaps in π activated by *p*5: Apply random feasible reassignments in ρ activated by *p*6: Set S←(π,ρ)7: Return *S*

The high-level execution logic for both the static and dynamic scenarios is supported by the notation summarized in Table 4. The general search process for the elective phase is described in Algorithm 5, while the dynamic rescheduling logic triggered by emergency arrivals is formalized in Algorithm 6.


**Algorithm 5** High-level execution logic for the discrete metaheuristic search in the elective scheduling scenario.
1: **Input:** Elective job set J, processing times D, resources, seed *s*2: Initialize population *P* with random schedules *S*3: **Evaluate:** For each S∈P, run simulator and compute fitness f(S)4: Set global best $S_{\mathrm{best}}\leftarrow \arg\min_{S\in P}f\left(S\right)$5: **while** termination budget not met **do**6:        **for** each candidate solution in *P* **do**7:                Apply algorithmic operators (Crossover/Mutation or Velocity/Position updates)8:                **if** method requires an explicit continuous-to-discrete mapping **then**9:                        Discretize/decode the internal state into a discrete schedule *S* (Figure 1)10:               **end if**11:               **Evaluate:** Run simulator to obtain fitness f(S)12:        **end for**13:        Update global best $S_{\mathrm{best}}$ if new minimum found14: **end while**15: **Return** Best schedule $S_{\mathrm{best}}$ and fitness $f(S_{\mathrm{best}})$



**Algorithm 6** Rescheduling control loop and “freeze-and-merge” logic for dynamic emergency integration.1:**Input:** Elective job set J, elective processing times D, emergencies E sorted by arrival time, seed *s*2:
**Phase 1: Initial elective schedule**
3:

$\left(\_,S_{cur}^{disc},\_,\_\right)\leftarrow RUNALGO\left(D,J,s\right)$

4:

$\left(\_,\_,S_{cur}^{det}\right)\leftarrow SIMULATE\left(S_{cur}^{disc},D\right)$

5:**for** each emergency E∈E arriving at $t_{E}^{arr}$ **do**6:        **Step 1: Identify system state (freeze criterion as in code)**7:
        $J^{S}\leftarrow \left\{j|\exists \;\mathrm{task}\;\mathrm{of}\;j\;\mathrm{in}\;S_{cur}^{det}\;\mathrm{with}\;Start<t_{E}^{arr}\right\}$8:        $J^{RS}\leftarrow \left(J\;\cup \;\{\mathrm{previous}\;\mathrm{emergencies}\;\mathrm{present}\;\mathrm{in}\;S_{cur}^{det}\}\right)\;\setminus \;J^{S}$9:        **Step 2: Construct re-optimization instance**10:        Sample emergency durations $D_{E}$ and define $D^{\prime }\leftarrow D{|}_{J^{RS}}\cup D_{E}$11:        *If* $j\in J^{RS}$*is a previous emergency not in* D, *reconstruct its operation times from* $S_{cur}^{det}$12:        Define new job list $J^{\prime }\leftarrow \left[E\right]+J^{RS}$13:        Update seed (as in code): $s^{\prime }\leftarrow s+\left\lfloor t_{E}^{arr}\right\rfloor +1000\cdot \left(\mathrm{emergency}\;\mathrm{index}\right)$
14:        **Step 3: Re-optimization (discrete encoding)**15:        $\left(\_,S_{new}^{disc},\_,\_\right)\leftarrow RUNALGO\left(D^{\prime },J^{\prime },s^{\prime }\right)$16:        Force emergency priority: move *E* to position 1 in the sequence of $S_{new}^{disc}$17:        **Step 4: Simulate and merge (merge acts on detailed schedules)**18:        $\left(\_,\_,S_{new}^{det}\right)\leftarrow SIMULATE\left(S_{new}^{disc},D^{\prime }\right)$19:        Keep frozen tasks from $S_{cur}^{det}$ for jobs in $J^{S}$20:        Merge remaining tasks from $S_{new}^{det}$, shifting times forward to satisfy:21:           (i) all merged tasks start at or after $t_{E}^{arr}$22:           (ii) room availability induced by frozen tasks23:           (iii) within-job precedence between operations24:        Update schedule $S_{cur}^{det}\leftarrow MERGEANDSHIFT\left(S_{cur}^{det},S_{new}^{det},J^{S},t_{E}^{arr}\right)$25:
**end for**
26:**Return** final detailed schedule $S_{cur}^{det}$


## 5. Experimental Setup

This section describes the experimental protocol implemented, including how stochastic instances are generated, how elective and emergency scenarios are simulated, how the algorithms are parameterized, and how performance is evaluated and compared statistically.

### 5.1. Data and Calibration

The experimental baseline comprises a set J of 15 elective surgeries derived from the dataset reported by Bai et al. [3]. Although originally collected in a different healthcare context, the selected procedures (e.g., Cholecystectomies) represent standard global clinical interventions with comparable operative structures. This dataset serves as a robust proxy for testing algorithmic adaptability and robustness prior to calibration with specific local Chilean data.

Each surgery j∈J requires a sequence of three operations. While the mathematical model in Section Table 1 uses a general index *i*, here we denote the specific simulation stages by o∈{1,2,3} (corresponding to APR, OR, and ARR) for explicit structural clarity. We define ${\bar{p}}_{j,o}$ as the nominal processing time and $p_{j,o}$ as the realized duration.

To capture operational variability, realized durations are sampled in every Monte Carlo replication via an independent Gaussian perturbation:

(11) $$p_{j,o}\leftarrow \max\left\{1,\;N\left({\bar{p}}_{j,o},\;{\left(0.15\;{\bar{p}}_{j,o}\right)}^{2}\right)\right\}.$$

This formulation ensures strictly positive values while introducing a 15% variability. Additionally, each surgery maps to a procedure type d∈{1,2,3} which determines auxiliary times; in this configuration, setup and cleanup are fixed at 30 and 20 min, respectively, for all types.

Emergencies are modeled as high-priority surgeries that arrive during the operating day, triggering an immediate rescheduling process. Each emergency is defined by an arrival time, a procedure type, and a maximum admissible start delay (set to 60 min). Processing times for these urgent cases are derived from a reference elective surgery of the same type, but with reduced variability (10%) to reflect the urgent nature of the procedure:

(12) $$p_{o}^{E}\leftarrow \max\left\{1,\;N\left({\bar{p}}_{ref,o},\;{\left(0.10\;{\bar{p}}_{ref,o}\right)}^{2}\right)\right\},\;o\in \left\{1,2,3\right\}.$$

This approach maintains realistic time scales while acknowledging the distinct stochastic profile of emergency cases.

### 5.2. Simulation Design

We perform a Monte Carlo study with 300 independent replications per scenario. In each replication, elective processing times are sampled first, and then each metaheuristic is executed to produce a solution that is evaluated by a discrete-event simulator. The simulator enforces precedence constraints between consecutive operations of the same surgery and capacity constraints on both rooms and personnel. Personnel assignment is dynamic: when an operation is ready to be scheduled, the simulator assigns the earliest-available qualified staff member from the corresponding pool; ties are broken deterministically to avoid introducing additional randomness.

The evaluation implements a blocking (no-buffer) behavior between stages: upstream resources may remain blocked until the downstream operation can start, capturing congestion effects in tightly coupled perioperative flows. All reported makespans therefore include setup and cleanup times and reflect blocking-induced delays whenever they arise.

#### 5.2.1. Emergency-Mode Rescheduling Policy

The simulation implements the dynamic rescheduling control loop defined in Section 3.2. As previously described, the system employs a *freeze-and-merge* strategy: upon the stochastic arrival of an emergency *E* at time $t^{arr}$, all operations started prior to this timestamp are fixed. The remaining elective operations are re-optimized along with *E* (inserted with high priority), and the resulting partial schedule is seamlessly merged to maintain consistency.

#### 5.2.2. Random Seeds and Reproducibility

Reproducibility is ensured by explicit seeding. For replication index *s*, elective-time sampling uses seed *s*. Emergency arrivals use seed 1000+s. During emergency integration, each rescheduling call uses a deterministic seed derived from the base seed and the emergency arrival time/index. Algorithm-level randomness is controlled by seeding both Python’s random generator and NumPy’s generator at the beginning of each run.

#### 5.2.3. Computing Environment

All experiments were executed on a Windows workstation equipped with an AMD Ryzen 5 9600 CPU, 32 GB RAM, and Python 3.13.7. Replications are parallelized using joblib; the number of worker processes is set in the runner as min(10,cpu_count−2). Reported runtimes correspond to this execution setup.

#### 5.2.4. Pseudo-Random Number Generators (PRNGs)

All stochastic components were controlled via explicit seeds. Elective and emergency sampling rely on NumPy’s legacy RNG interface, which uses the MT19937 (Mersenne Twister) bit generator through RandomState. Algorithmic randomness is also seeded via Python’s random module, which likewise implements a Mersenne Twister PRNG.

### 5.3. Benchmark Instances

The benchmark defines the fixed resource capacities constrained by the elective workload described in Section 5.1. The system includes 4 rooms per stage (12 rooms total) and three personnel pools, one per stage, each containing 6 staff members.

The elective day contains 15 surgeries (‘jobs’) selected from three representative outpatient procedures reported in [3]: Laparoscopic Cholecystectomy (Type 1), Open Cholecystectomy (Type 2), and Common Bile Duct Exploration (Type 3). Table 5 details the nominal duration profile and clinical type for each job.

In emergency mode, we inject 2 emergencies per replication. Emergency arrival times are generated from a truncated normal distribution:

(13) $$t^{arr}∼\mathrm{clip}\left(N(450,\;150^{2}),\;100,\;800\right),$$

which ensures that arrivals occur during the operative horizon rather than after completion of the elective plan.

### 5.4. Performance Metrics

Algorithm performance is assessed primarily through makespan and secondarily through runtime. Let $C_{\max}$ denote the completion time of the last finished task in the simulated schedule (equivalent to $x_{\tau }$ in the mathematical model). For each algorithm, we report the minimum, median, mean, and standard deviation of $C_{\max}$ across replications.

Internally, the metaheuristics minimize a composite fitness function *f* that extends the original objective defined in Equation (1). While the MIP model strictly minimizes makespan under hard constraints, the metaheuristics incorporate auxiliary terms, specifically patient waiting times and resource imbalance, to distinguish between solutions with equal makespans and guide the search toward operationally robust schedules.

Table 6 summarizes the coefficients used in this augmented objective function and the fixed operational parameters of the simulation.

The fitness function *f* is defined as:

(14) $$f\;=\;\underset{\mathrm{Original}\;\mathrm{Objective}\;(\mathrm{Eq}.\;1)}{\underset{︸}{C_{\max}\;+\;\alpha \sum_{J\in J}\sum_{i\in I_{J}}\sum_{r\in R_{i}}x_{J_{i}}^{r}\;z_{J_{i}}^{r}}}\;+\;\underset{\mathrm{Auxiliary}\;\mathrm{Quality}\;\mathrm{Metrics}}{\underset{︸}{\beta \sum_{J\in J}W_{J}\;+\;\gamma \max_{J\in J}W_{J}\;+\;\delta \;\Psi }},$$

where $x_{J_{i}}^{r}$ corresponds to the processing start time (after setup) on the assigned room, $W_{J}$ represents the internal waiting time of surgery *J* between stages, and Ψ quantifies the resource load imbalance.

#### 5.4.1. Imbalance Term Ψ

Let $u_{r}$ be the number of assigned operations executed on room *r*. For each room group *G* (APR, OR, ARR), we compute the coefficient of variation and apply a progressive penalty for unused rooms:

(15) $$\mu_{G}=\frac{1}{\left|G\right|}\sum_{r\in G}u_{r},\;\sigma_{G}=\sqrt{\frac{1}{\left|G\right|}\sum_{r\in G}{\left(u_{r}-\mu_{G}\right)}^{2}},\;{\mathrm{CV}}_{G}=\frac{\sigma_{G}}{\mu_{G}}.$$

Here, $\mu_{G}$ and $\sigma_{G}$ denote the mean and standard deviation of ${\left\{u_{r}\right\}}_{r\in G}$, respectively, and ${\mathrm{CV}}_{G}$ is the corresponding coefficient of variation.

The total imbalance is defined as $\Psi =\sum_{G\in \{APR,OR,ARR\}}\left({\mathrm{CV}}_{G}+k_{G}\left(k_{G}+1\right)\right)$, where $k_{G}$ is the count of unused rooms in group *G*.

#### 5.4.2. Emergency Integration Metrics

To quantify the responsiveness of the system, we define the *Integration Delay* ($D_{int}$) for an emergency surgery *E*. This metric measures the temporal gap between the stochastic arrival time ($t_{E}^{arr}$) and the actual setup start time of its first operation ($x_{E_{1}}^{r}$). It is calculated as:

(16) $$D_{int}\left(E\right)=x_{E_{1}}^{r}-t_{E}^{arr}$$

Additionally, we report the specific Arrival Time and Integration Time for representative cases.

### 5.5. Metaheuristic Configuration

We evaluate four population-based metaheuristics: GA, dPSO, SBOA, and dMShOA. To ensure a fair comparison, all methods share a common termination criterion and population size. Table 7 details the specific hyperparameters and search space bounds used in the experiments.

All methods optimize the same encoding: a priority list for surgeries and a per-operation room assignment. The continuous domains in SBOA and dMShOA are discretized using the mechanisms described in Section 4.2.

## 6. Experimental Results and Analysis

This section details the quantitative findings obtained following the execution of 300 independent Monte Carlo replications for each of the four metaheuristic algorithms evaluated: the GA, dPSO, SBOA, and dMShOA. The experiments were conducted under the $S_{emergency}$ modality, which introduces the stochastic arrival of urgent cases and forces dynamic sequential rescheduling.

### 6.1. Global Schedule Efficiency (Makespan)

The primary performance metric is the *makespan* ($C_{max}$), defined as the completion time of the last surgical operation within the planning horizon, including cleaning times and delays induced by blocking. Table 8 provides a structured breakdown of the performance metrics across the evaluated metaheuristics. Each row corresponds to a specific algorithm, while the columns enumerate critical statistical descriptors: the minimum observed value (Min), central tendency measures (Median and Mean μ), dispersion indicators (Standard Deviation σ and Coefficient of Variation CV), and the average computational overhead (CPU Time in seconds).

**Figure 2:**
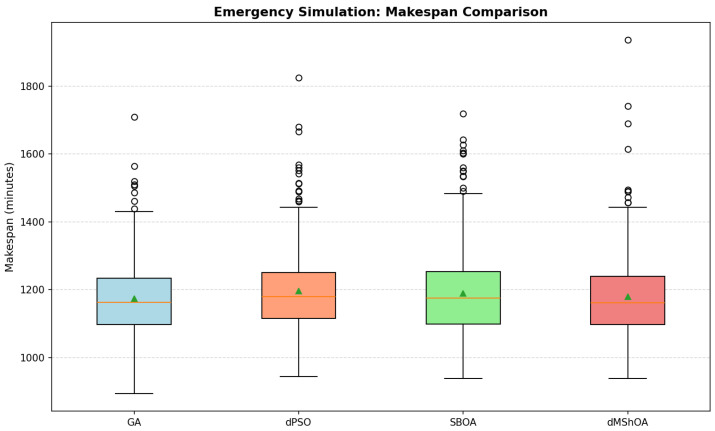
Comparative makespan distributions for the evaluated metaheuristics across 300 Monte Carlo replications, illustrating the impact of stochastic emergency arrivals.

To visualize the distributional characteristics and the impact of stochastic uncertainty on the surgical schedule, Figure 2 illustrates the makespan distribution using box plots for the 300 replications per method. In this representation, the central box encompasses the Interquartile Range (IQR), with the internal orange horizontal line indicating the median and the green triangle denoting the arithmetic mean. The vertical lines extend to the most extreme data points within the standard range, while open circles represent outliers, corresponding to instances where specific emergency arrival patterns significantly extended the schedule duration.

Evaluation of central tendencies and peak optimization capacities establishes a definitive performance hierarchy among the evaluated metaheuristics. The minimum makespan (Min) serves as a critical proxy for the algorithm’s potential to identify near-optimal schedules under ideal stochastic conditions. The GA attained the absolute minimum of 894.04 min, significantly outperforming dMShOA (938.24 min) and SBOA (938.38 min).

This disparity indicates that the GA provides superior deep-exploration capabilities, enabling access to high-quality regions of the search space that remain unreachable by alternative methods under stochastic pressure. However, a nuanced finding emerges from the comparison of means and medians: while the GA maintains the best mean performance (1174.12 min), dMShOA achieved the lowest median (1161.26 min), marginally surpassing the GA median of 1162.70 min. As evidenced in Figure 2, this discrepancy is explained by the heavy right tail of the dMShOA distribution. The presence of high-magnitude outliers (reaching up to approximately 1950 min) penalizes its average, despite the algorithm achieving exceptionally competitive results in over 50% of the cases.

The dispersion metrics, σ and CV, quantify algorithmic robustness against the uncertainty of surgical times and emergency arrivals. GA presented the highest operational predictability with the lowest variability (σ=114.30), whereas SBOA showed the highest dispersion (σ=132.36). Finally, regarding computational efficiency, GA also proved to be the most agile (12.93 s), whereas SBOA required nearly 85% more time (23.99 s) to complete the same number of replications.

#### Distributional Analysis of Makespan

**Figure 3:**
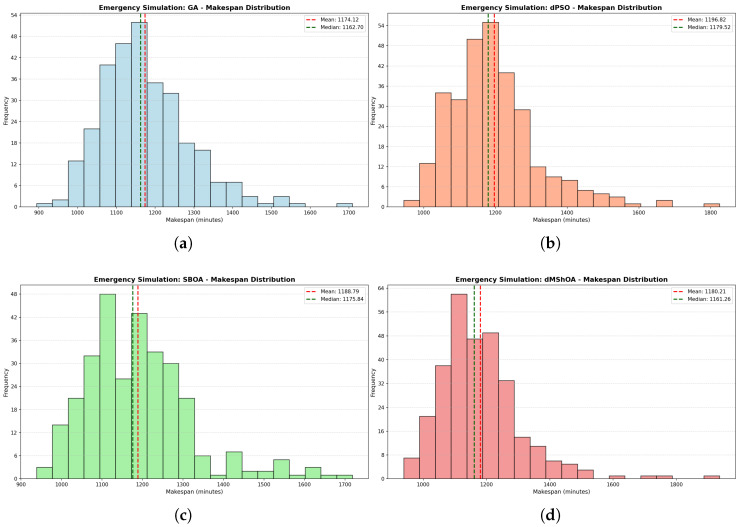
(**a**) GA Distribution. (**b**) dPSO Distribution. (**c**) SBOA Distribution. (**d**) dMShOA Distribution.

To provide a deeper understanding of the algorithmic behavior under stochastic pressure, Figure 3 presents the frequency distribution of the makespan across the 300 Monte Carlo replications for each metaheuristic. The figure is composed of four individual histograms (a–d), where the horizontal axis represents the makespan duration in minutes and the vertical axis denotes the frequency of occurrence. Each plot includes two distinctive vertical reference lines: a red dashed line indicating the arithmetic mean (μ) and a green dashed line marking the median.

The morphological analysis of the histograms reveals that all four algorithms exhibit a positive skewness (right-skewed distribution), which is a direct consequence of the $S_{emergency}$ modality. The stochastic arrival of urgent cases occasionally forces significant rescheduling, creating the “heavy tails” observed in the plots. However, the degree of concentration varies significantly between methods.

The dMShOA (Figure 3d) displays the highest peak frequency, with more than 60 observations concentrated in the 1100–1150 min range. This indicates a high degree of convergence and consistency in standard scenarios, despite being the method most affected by extreme outliers that reach up to 1800 min. In contrast, the GA distribution (Figure 3) shows a more balanced and centered profile, with its mean (1174.12) and median (1162.70) being the closest among the set. This symmetry suggests that GA is more robust and less susceptible to the drastic shifts in makespan caused by random seeds.

Conversely, dPSO (Figure 3b) and SBOA (Figure 3c) exhibit flatter distributions with a wider spread. The dPSO histogram is clearly shifted toward the right, confirming its overall lower efficiency as its primary density occurs at higher makespan values. SBOA shows a multi-modal tendency in its central bins, which correlates with the high standard deviation (σ=132.36) previously identified, signifying a higher sensitivity to the initial conditions of each simulation instance.

### 6.2. Computational Efficiency and Scalability

**Figure 4:**
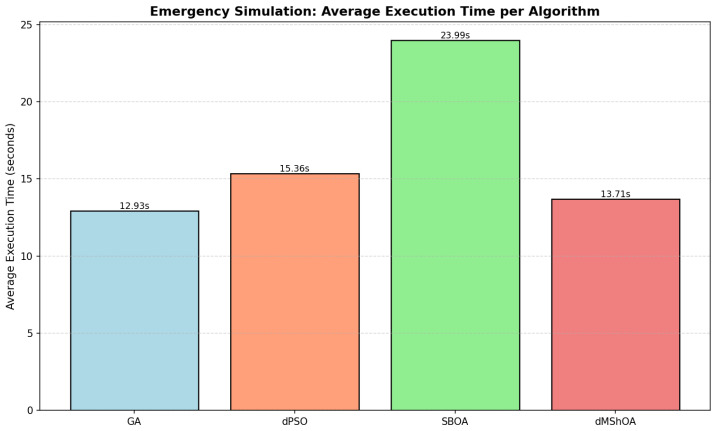
Mean computational overhead (seconds) required for full simulation instances, including initial elective planning and sequential rescheduling for emergency integration. The labels above each bar indicate the mean temporal overhead for a complete simulation instance.

In dynamic surgical environments governed by the $S_{emergency}$ modality, computational efficiency is as critical as schedule quality. The ability to generate a revised schedule in near real-time following the stochastic arrival of an urgent case is a prerequisite for clinical viability. Figure 4 presents a comparative analysis of the average execution times (CPU time) required by each metaheuristic to complete a full simulation instance, encompassing both the initial elective planning and the subsequent dynamic rescheduling events.

The visualization in Figure 4 utilizes a bar chart where the vertical axis represents time in seconds and the horizontal axis identifies the algorithms, maintaining the color coding used in previous distributional analyses. As reported in Table 8, the GA proved to be the most computationally efficient method, with a mean execution time of 12.93 s. This is closely followed by the dMShOA, which required 13.71 s. The 6% difference between these two metaheuristics is considered negligible in an operational hospital setting, positioning dMShOA as a highly competitive alternative due to its superior median performance in makespan.

A significant disparity is observed when evaluating the SBOA. As formulated in Equation (17), the computational demand of SBOA is substantially higher than that of the leading method:

(17) $$T_{exec}\left(SBOA\right)\approx 1.85\times T_{exec}\left(GA\right)$$

With a mean execution time of 23.99 s, SBOA exhibits an 85.5% increase in CPU overhead compared to GA. This performance gap is attributed to the internal complexity of the SBOA search operators, which involve multi-phase transitions between exploration and “attack” (exploitation) mechanisms. While a 24-s response time remains within the acceptable threshold for a single rescheduling event, this cumulative overhead could limit the algorithm’s scalability in larger instances or in scenarios requiring hundreds of rapid Monte Carlo iterations for decision support.

Finally, the dPSO algorithm occupies an intermediate position with a mean of 15.36 s. While faster than SBOA, its lack of competitiveness in makespan efficiency (as analyzed in Section 6.1) makes it a less attractive option for this specific emergency surgical context. Consequently, GA and dMShOA emerge as the most balanced solutions, providing a superior trade-off between optimization convergence and computational agility.

### 6.3. Convergence Analysis

**Figure 5:**
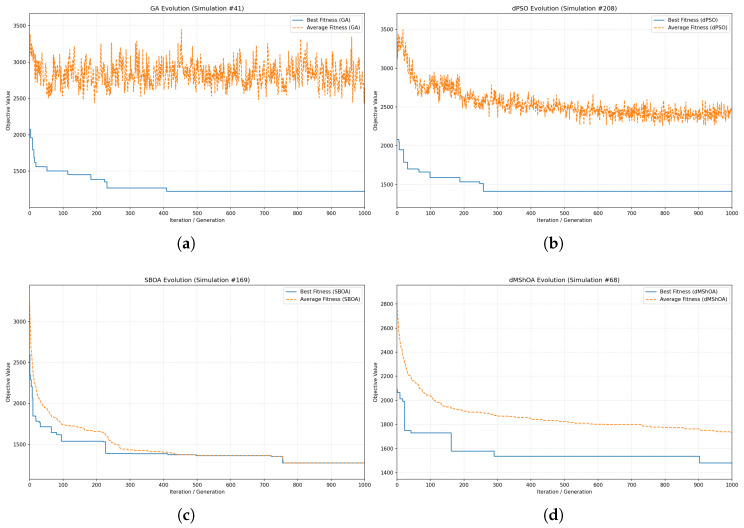
(**a**) GA Convergence. (**b**) dPSO Convergence. (**c**) SBOA Convergence. (**d**) dMShOA Convergence.

The search efficiency and the balance between exploration and exploitation are further examined through the convergence profiles of the evaluated metaheuristics. Figure 5 illustrates the evolution of the objective value (Makespan) over 1000 iterations for a representative simulation instance of each algorithm. Each plot displays the *Best Fitness* (solid blue line), representing the global optimum found by the population up to that point, and the *Average Fitness* (dashed orange line), which reflects the overall state of the population in the search space.

A primary observation in the convergence behavior is the distinct morphology of the average fitness lines. In the GA (Figure 5a) and dPSO (Figure 5b), the average fitness exhibits a highly erratic and noisy behavior. In GA, this phenomenon is a direct consequence of the stochastic operators of crossover and mutation. High mutation rates, necessary to prevent premature convergence in complex SS, continuously introduce new individuals into the population that may have low fitness, thus keeping the population average fluctuating. This “noise” is an indicator of healthy population diversity, allowing GA to escape local optima and eventually reach the absolute minimum observed in earlier sections.

Conversely, the SBOA (Figure 5c) and dMShOA (Figure 5d) demonstrate a markedly “smoother” decay in their average fitness. In SBOA, this behavior reflects a high degree of swarm coordination; as the iterations progress, the “secretary birds” converge cohesively toward the leading solution, reducing the variance within the population. While this results in a stable and monotonic convergence, it also increases the risk of the population collapsing into a local optimum if the initial exploration phase is insufficient.

The dMShOA presents a balanced profile, where the best fitness decreases in significant steps, indicating successful “jumps” to better regions of the search space, while the average fitness follows a smooth, controlled trajectory. This suggests that dMShOA maintains enough diversity to improve the best solution while ensuring that the population as a whole is effectively exploiting the most promising areas. In contrast, the dPSO profile shows long plateaus in the best fitness, confirming its slower convergence rate and explaining its overall lower efficiency in the 1200-minute range compared to the other evaluated methods.

### 6.4. Emergency Integration Analysis

One of the most significant contributions of this study is the evaluation of algorithmic responsiveness under stochastic disruption. Unlike elective planning, emergency integration requires the metaheuristic to reconcile the existing schedule with the immediate need for surgical intervention. To quantify this capability, we define the *Integration Delay* ($D_{int}$) for an emergency surgery E∈E. This metric represents the temporal gap between its arrival time ($t_{E}^{arr}$) and the scheduled start time of its first operation (i=1) on the assigned resource *r* ($x_{E_{1}}^{r}$), as formulated in Equation (18):

(18) $$D_{int}\left(E\right)=x_{E_{1}}^{r}-t_{E}^{arr}$$

In our experimental framework, E16 and E17 denote the two emergency identifiers injected per replication. The clinical type of each emergency is sampled stochastically, and its durations are generated by sampling around a reference elective profile with a 10% standard deviation. Table 9 reports the reference profiles used for this parameterization, providing the necessary clinical context for the procedures analyzed.

Table 10 summarizes the latency metrics for E16 and E17, extracted from the best recorded schedule of each metaheuristic. Since the optimal solution for each algorithm stems from a distinct Monte Carlo replication, the specific arrival instants $t_{E}^{arr}$ vary naturally across methods.

The realized net processing times ($p_{E,o}$) observed in those specific best schedules are reported in Table 11. These values reflect the actual work time excluding setup and cleanup, highlighting the diverse clinical scenarios the algorithms resolved in their respective optimal runs.

#### Responsiveness and Clinical Resilience Analysis

The results show that algorithmic performance depends strictly on the system’s state at arrival. SBOA outperformed others during early and mid-phases; for E16, it achieved instantaneous integration ($D_{int}=0$) by exploiting gaps without displacing elective procedures. Even for E17, its 8.47-min delay remained well below clinical thresholds.

In contrast, dMShOA struggled with system saturation. For E17 (arriving at minute 346.19), the delay reached 81.37 min, exceeding the 60-min safety threshold for urgent trauma.

GA adapted best to late-stage arrivals: despite a 30.35-min delay for E16, it reached perfect integration ($D_{int}=0$) for E17 (minute 642.08). This confirms GA’s global search strength in re-optimizing the schedule “tail.” Conversely, dPSO was inconsistent, matching SBOA in E16 but failing in E17 with a 79.86-min latency.

### 6.5. Statistical Significance and Comparative Validation

To rigorously validate the performance hierarchy observed in the descriptive analysis, we perform an inferential statistical contrast. The objective is to determine if the differences in makespan performance are statistically significant or if the algorithms remain within a competitive efficiency frontier.

We formulate the following hypothesis test for each pair of algorithms, where the value 0.5 represents the threshold of stochastic dominance:

- **Null Hypothesis ($H_{0}$):** The algorithms are statistically competitive or equivalent, meaning the probability that a random makespan from algorithm *A* is lower than one from algorithm *B* is equal to or greater than 0.5.
  (19) $$H_{0}:P\left(C_{max,A}<C_{max,B}\right)\geq 0.5$$
- **Alternative Hypothesis ($H_{1}$):** There is a statistically significant difference in the distributions, indicating that algorithm *A* is outperformed by algorithm *B*.
  (20) $$H_{1}:P\left(C_{max,A}<C_{max,B}\right)<0.5$$

Given the non-normal nature and the presence of outliers in the surgical data, we utilized the non-parametric Mann-Whitney *U* test with a significance level of α=0.05. The decision rule is strictly defined as follows: if the resulting value is ≥0.5, no significant difference exists, indicating that the algorithms are statistically competitive. Conversely, if the value is <0.5, a significant performance gap is confirmed.

Table 12 presents the pairwise comparisons between the metaheuristics. This table consists of six columns: the first identifies the pair of algorithms compared (following the order GA, dPSO, SBOA, and dMShOA); the second and third report the *U* statistic and the *p*-value; the fourth and fifth detail the effect size through the rank-biserial correlation (*r*) and its magnitude; and the final column confirms the significance. For all competitive results where no statistical difference exists, the *p*-value is explicitly represented as “≥0.05”.

The statistical analysis yields a fundamental finding regarding the competitiveness of the bio-inspired suite. The results confirm that the GA maintains a statistically validated advantage over the dPSO (p=0.0239). Although the effect size (r=0.11) is small, the consistency of this gap across 300 replications indicates that the evolutionary operators in GA provide a more robust exploration of the search space compared to the discrete particle swarm mechanics in this specific surgical context.

Most importantly, the comparisons between GA, SBOA, and dMShOA show no statistically significant differences (p≥0.05). This has a profound implication: the recently introduced metaheuristics, SBOA and dMShOA, have successfully reached the efficiency frontier established by the GA baseline. These three algorithms behave as statistically equivalent competitors in terms of average makespan efficiency.

Consequently, the choice between GA, SBOA, and dMShOA should not be dictated solely by makespan performance, but by secondary operational criteria. Through this statistical analysis, we have clarified that while these algorithms reach the same statistical efficiency frontier, our proposed discretized versions of SBOA and dMShOA do so with significantly greater stability in their search trajectories. We observed that the erratic path of the GA is a direct byproduct of the exploratory mutation strategy we implemented to navigate the rugged search landscape. In contrast, our discretized bio-inspired metaheuristics demonstrate a more cohesive swarm convergence, proving that they can reach high-quality solutions with a more predictable and stable optimization process, which is essential for reliable decision support in clinical environments.

### 6.6. Structural Analysis of Optimal Scheduling Strategies

While statistical descriptors provide a global view of performance, visual inspection of the Gantt charts allows for a qualitative assessment of resource allocation logic and structural robustness. To characterize the “efficiency frontier” of each metaheuristic, Table 13 summarizes the metrics for the absolute best-case scenarios identified across the 300 Monte Carlo replications.

This table acts as a benchmark for peak optimization capacity: the rows identify the evaluated metaheuristics, while the columns enumerate the minimum makespan ($C_{max}$) achieved, the average computational overhead (CPU time), and a qualitative classification of the resulting scheduling strategy.

#### 6.6.1. Genetic Algorithm Strategy

**Figure 6:**
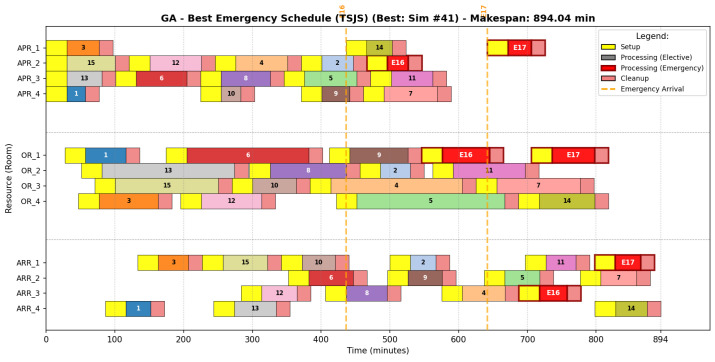
Representative Gantt chart for the GA optimal schedule (Simulation #41).

The GA schedule (Figure 6) represents the highest degree of efficiency achieved in this study. In this visualization, the vertical axis denotes the physical resources (rooms) categorized by stage (APR, OR, ARR), while the horizontal axis tracks the temporal progression in minutes. The GA strategy is characterized by extreme temporal compaction and high synchronization between stages.

The underlying sequencing pattern, detailed in Table 14, highlights a “just-in-time” alignment. For instance, in OR_1, GA manages to sequence five procedures, including both stochastic emergencies (E16 and E17), at the end of the operative block. By effectively managing the ARR bottleneck, evidenced by the continuous occupancy in ARR_1 through ARR_3, the GA ensures that patients transition seamlessly from surgery to recovery, thereby preventing back-blocking effects.

#### 6.6.2. Discrete Particle Swarm Optimization Strategy

**Figure 7:**
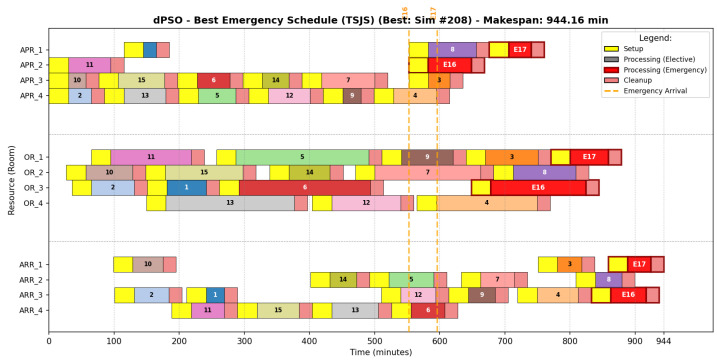
Representative Gantt chart for the dPSO schedule (Simulation #208).

The dPSO strategy (Figure 7) exhibits the most dispersed allocation pattern. Visual inspection reveals significant idle periods in the APR stage, particularly between minutes 200 and 400. This dispersion is confirmed by the sequence in Table 15, where the workload is unevenly distributed; while OR_3 is tasked with six procedures, OR_4 remains largely underutilized with only one surgical case. This imbalance propagates delays throughout the system, leading to a higher makespan and poor resilience against emergency-induced disruptions.

#### 6.6.3. Secretary Bird Optimization Strategy

**Figure 8:**
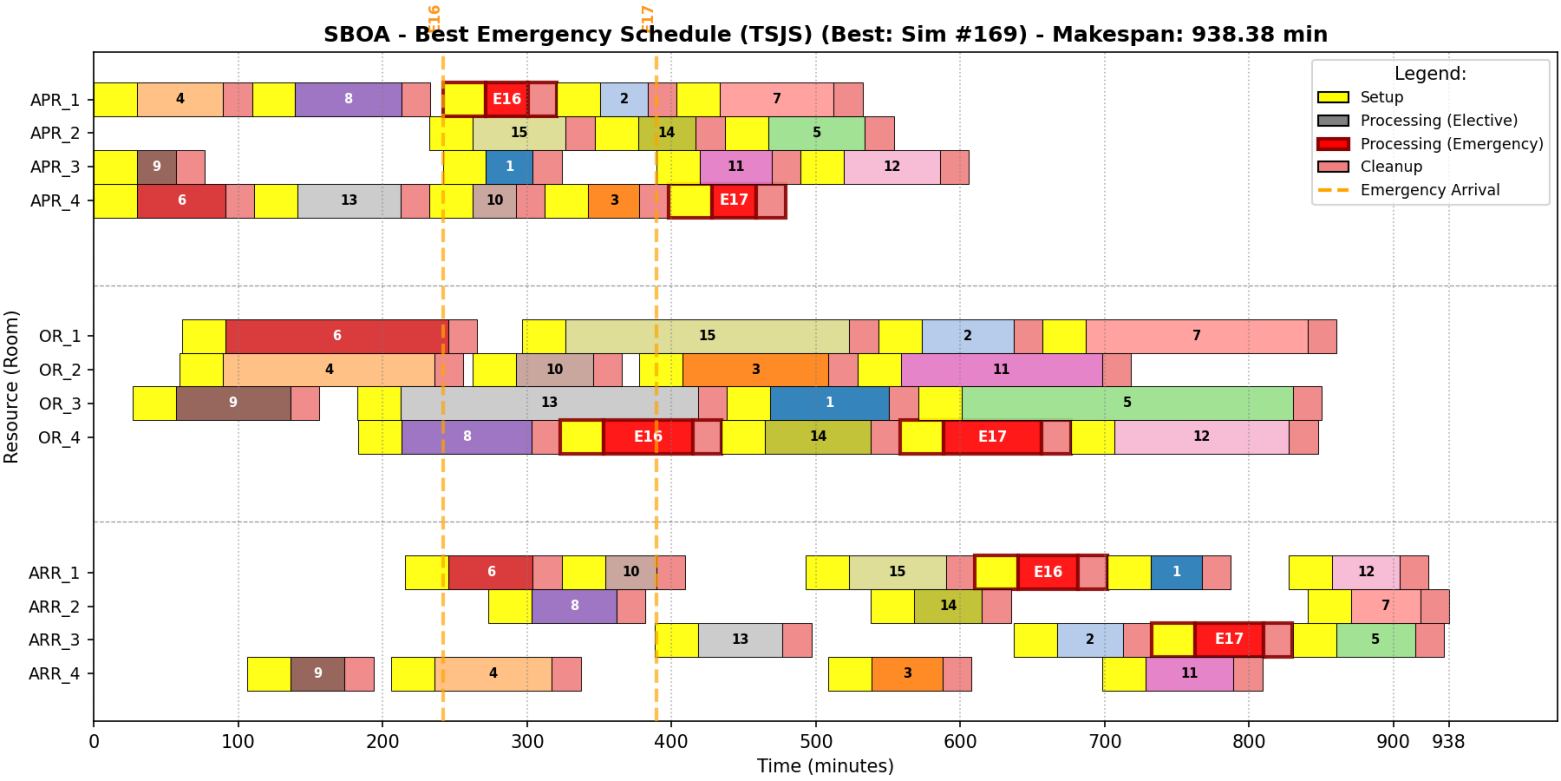
Representative Gantt chart for the SBOA schedule (Simulation #169).

The SBOA schedule (Figure 8) reflects a strategy of Adaptive Flexibility. Unlike the aggressive compaction of GA, SBOA maintains a “looser” resource map. The sequencing detailed in Table 16 shows a highly symmetrical load distribution across operating rooms (distributed as 4, 4, 4, and 5 operations per room, respectively). This symmetry creates natural capacity buffers, allowing the system to absorb stochastic arrivals such as E16 and E17 in OR_4 without causing the “hydraulic locking” or cascading delays seen in more rigid methods.

#### 6.6.4. Discrete Mantis Shrimp Optimization Strategy

**Figure 9:**
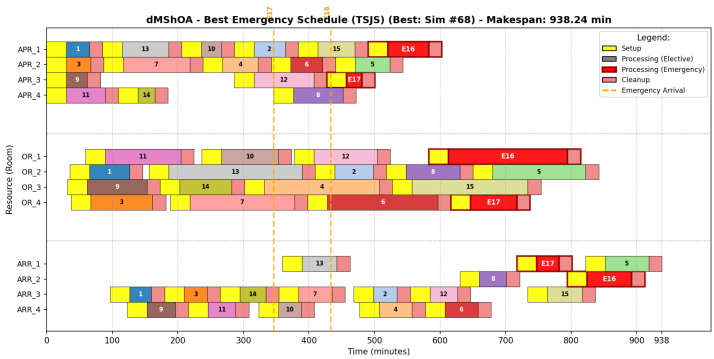
Representative Gantt chart for the dMShOA optimal schedule (Simulation #68).

Despite its competitive makespan, the dMShOA strategy reveals structural vulnerabilities (Figure 9). Visual analysis shows instances of OR Fragmentation, particularly in OR_1 and OR_4. As shown in Table 17, dMShOA distributes emergencies across different rooms to reduce team pressure.

However, this logic leads to a higher susceptibility to “chain blocking”. By prioritizing local flow optimization, the algorithm saturates recovery beds prematurely, as evidenced by the sequence of seven elective patients in ARR_3, thereby restricting the system’s maneuverability when faced with urgent insertions.

## 7. Conclusions and Future Work

This investigation provided a rigorous assessment of four population-based metaheuristics applied to a dynamic three-stage SS model encompassing anesthesia preparation, operative procedures, and post-anesthesia recovery. By simulating stochastic emergency arrivals and enforcing a no-buffer blocking policy, we identified critical performance trade-offs that extend beyond traditional makespan minimization. Our results confirm that while the GA remains a robust and fast baseline for global efficiency, our proposed discretized versions of SBOA and MShOA have successfully reached the same statistical efficiency frontier, performing as equivalent competitors in terms of average makespan optimization.

In accordance with the No Free Lunch Theorem [36] we have demonstrated that providing algorithmic diversity is essential for complex hospital management landscapes. We found that no single metaheuristic is universally superior; while the GA achieves high temporal compaction through aggressive exploration, our discretized bio-inspired proposals offer a much more stable and cohesive convergence path. This stability is crucial for clinical decision-support systems, as it provides more predictable optimization trajectories without sacrificing the quality of the final schedule. By successfully adapting these modern algorithms to a discrete domain, we have expanded the computational toolkit available for managing highly constrained surgical environments.

Beyond numerical equivalence, the analysis revealed a fundamental trade-off between static efficiency and dynamic responsiveness, characterized here as a flexibility paradox. This study identified a critical systemic failure defined as hydraulic locking, wherein the saturation of recovery units precipitates a backward-propagating obstruction that paralyzes upstream operating rooms. In this context, the Genetic Algorithm achieved operational robustness through aggressive schedule compaction and high predictability, making it particularly suitable for personnel planning due to its low variability. Conversely, the SBOA approach demonstrated a form of passive robustness; by maintaining a naturally balanced resource distribution with capacity buffers, it ensured near-zero integration latency for urgent cases, suggesting a clear superiority for trauma centers or facilities requiring immediate emergency access.

From a public health management perspective, the socio-economic impact of these algorithms is substantial. Given that surgical services typically consume approximately 40% of total hospital operating expenditures, the efficiency gains identified, amounting to a recovery of roughly 90 annual operating hours per theater, represent a 6.7% increase in potential capacity. In the context of the Chilean public health crisis, this optimization acts as a “virtual expansion” of infrastructure, providing the necessary scheduling margin to address the Non-GES surgical backlog, which currently exceeds 400,000 patients according to national reports [21,22], without requiring new physical facilities. While in a real-world clinical setting this margin may act as an operational buffer against friction and fatigue, theoretically it offers the scheduling space to facilitate between 47,000 and 63,000 additional procedures per year across the national network, providing a concrete and scalable mechanism to mitigate the substantial Non-GES surgical backlogs within the Chilean public health system. By significantly reducing inter-stage waiting times and preventing resource saturation, these metaheuristics offer a viable pathway to enhance both institutional productivity and the clinical quality of patient care.

Regarding the implementation of this model in real-world clinical environments, the framework is designed to be infrastructure-agnostic, focusing on the efficient orchestration of existing resources rather than requiring structural changes to the hospital’s physical layout. The primary requirement for deployment is a digital interface with the Electronic Health Record (EHR) to feed the stochastic parameters. As our results indicate, the computational overhead is minimal (with rescheduling occurring in under 25 s—meaning the model can run on standard hospital hardware and existing computing assets. This eliminates the need for specialized or high) performance servers, making the implementation a matter of data interoperability and digital transformation. Such an approach aligns with current modernization efforts in the Chilean healthcare network to maximize installed capacity without further capital expenditure.

The final stage of this research involves a critical transition from controlled simulations to empirical calibration. Future work will focus on validating this optimization framework using real-world operational data from a public hospital in the Los Lagos Region of Chile. This collaborative phase will allow the model to reflect specific regional resource constraints and local stochastic demand patterns, ensuring that the resulting decision-support tool is precisely tailored to the demographic and clinical realities of the Chilean healthcare infrastructure. Establishing this methodological baseline represents a decisive step toward the digital transformation of surgical management, effectively bridging the gap between theoretical combinatorial optimization and frontline clinical governance.

## Figures and Tables

**Table 1:** Mathematical notation, sets, and parameters used in the resilient three-stage flexible flow shop model.

**Sets and Indices**	**Description**
*J*	Set of elective surgeries (jobs); j∈J.
E	Set of emergency surgeries (stochastic arrivals); E∈E.
$J^{RS}$	Set of reschedulable pending jobs; $Q\in J^{RS}$ (where Q≠j).
*R*	Set of physical resources (rooms); r,u∈R.
*I*	Set of operational stages (APR, OR, ARR); i,k∈{1,2,3}.
$I_{last}$	Subset representing the final operational stage (i=3).
*P*	Set of human resources (surgeons and staff); p∈P.
*D*	Set of clinical procedure types; d∈D.
**Parameters**	**Description**
$t_{i_{dp}}^{r}$	Processing time of operation *i* on resource *r* for procedure type *d* and surgeon *p*.
$t_{i}^{s},t_{i}^{c}$	Required setup and cleaning times for stage *i*, respectively.
$t_{E}^{arr}$	Stochastic arrival time of emergency surgery *E*.
$b_{i}$	Maximum admissible waiting or stay time allowed in stage *i*.
$e^{r}$	Earliest availability time of resource *r* after a rescheduling event.
*H*	A sufficiently large positive constant (Big M).
α,ω	Weighting factors for elective early starts and emergency priority, respectively.
τ	Dummy completion operation used for makespan calculation.
**Decision Variables**	**Description**
$z_{j_{i}}^{r}$	Binary: 1 if resource *r* is assigned to operation *i* of surgery *j*; 0 otherwise.
$\theta_{ru}^{j}$	Binary: 1 if resource *r* is used before resource *u* in surgery *j*; 0 otherwise.
$y_{r}^{jQ}$	Binary: 1 if surgery *j* is scheduled before surgery *Q* on resource *r*; 0 otherwise.
$x_{j_{i}}^{r}$	Scheduled start time of operation *i* of surgery *j* on resource *r*.
$L_{j_{i}}^{r}$	Completion time of operation *i* of surgery *j* on resource *r*.
$x_{\tau }$	Total makespan of the daily surgical plan.

**Table 2:** Translation of Key Algebraic Constraints into Clinical and Operational Protocols.

Constraint	Algebraic Form	Clinical/Operational Translation	Primary Goal
Precedence	Equation (2)	Clinical Path Continuity: A surgery must finish one stage (e.g., OR) before the next (ARR) begins.	Patient Safety
Exclusivity	Equation (3)	Personnel Exclusivity: A surgeon or specialized nurse can only be assigned to one patient at a time.	Error Reduction
Sanitation	Equation (4)	Turnover Protocols: Mandatory cleaning and sterilization cycles required between procedures.	Infection Control
Blocking	Equation (5)	Operating Room Holding: The OR remains occupied (blocked) if no bed is available in the recovery unit.	Flow Management
Safety Window	Equation (8)	Triage Responsiveness: High-priority emergency procedures must begin within a set clinical safety window.	Risk Mitigation

**Table 3:** Additional notation for operator-level pseudocode (Algorithms 1–4).

Symbol	Meaning
π	Priority list (job permutation) in the discrete encoding.
ρ	Room-assignment map (per job and stage) in the discrete encoding.
*N*	Population/swarm size.
$p_{c},\;p_{m}$	Crossover and mutation probabilities (GA).
$E_{\mathrm{elite}}$	Elitism count (GA).
$V^{\pi },\;V^{\rho }$	Continuous velocities for sequence and room components (dPSO).
$S^{pbest}$	Personal-best encoding of a particle (dPSO).
σ(·)	Sigmoid mapping to activation probability.
$X\in {\left[L,U\right]}^{d}$	Continuous position vector and search domain (SBOA/dMShOA).
Δ	Velocity-like displacement vector used to derive transition probabilities (dMShOA).
*k*	Movement magnitude control parameter (dMShOA).

**Table 4:** Summary of parameters and operational logic notation utilized in the high-level execution of Algorithms 5 and 6.

Symbol/Identifier	Meaning
Common (both algorithms)	
J	Set of elective surgeries (jobs).
D	Realized processing-time dictionary used by the simulator for the current replication (net operation times per job and stage).
E	List/set of emergency surgeries injected in emergency mode.
*E*	A specific emergency surgery (job), E∈E.
$t_{E}^{arr}$	Arrival time of emergency *E* (minutes).
*s*	Base random seed for the replication.
f(S)	Fitness value computed by the unified discrete-event simulator.
$S_{\mathrm{best}}$	Best discrete solution found by the metaheuristic within its termination budget.
Termination budget	Fixed iteration/generation budget.
*Discrete vs. detailed schedules (emergency mode pseudocode)*	
$S^{disc}$	Discrete solution encoding evaluated by the simulator.
$S_{cur}^{disc}$	Current discrete solution encoding used to generate $S_{cur}^{det}$ via simulation (elective phase).
$S_{new}^{disc}$	Discrete solution encoding produced by re-optimizing the combined instance $J^{\prime }$.
$S^{det}$	Detailed schedule returned by the simulator (task list).
$S_{cur}^{det}$	Current detailed schedule (task list) before integrating the next emergency.
$S_{new}^{det}$	New detailed schedule obtained by simulating the re-optimized discrete solution for the combined instance $J^{\prime }$.
RunAlgo (D,J,s)	Call to a metaheuristic (GA, dPSO, SBOA, dMShOA).
Simulate $\left(S^{disc},D\right)$	Discrete-event simulation/evaluation procedure that returns $f(S^{disc})$ and, when requested, a detailed schedule $S^{det}$.
Start	In $S^{det}$, timestamp at which an operation begins its *setup* on the assigned room.
ProcessingEnd	In $S^{det}$, timestamp at which net processing finishes.
Finish	In $S^{det}$, timestamp at which cleanup ends and the room becomes available.
*Emergency-mode sets used in rescheduling*	
$J^{S}$	Frozen (started) jobs at time $t_{E}^{arr}$: jobs for which *any* task in $S_{cur}^{det}$ satisfies Start $<t_{E}^{arr}$.
$J^{RS}$	Reschedulable jobs: current jobs (electives plus previously injected emergencies) excluding $J^{S}$.
$J^{\prime }$	Combined job list submitted to re-optimization: $\left[E\right]+J^{RS}$.
$D_{E}$	Emergency operation durations generated for *E*.
$D^{\prime }$	Combined durations dictionary used for re-optimization/simulation of $J^{\prime }$: $D^{\prime }=D{|}_{J^{RS}}\cup D_{E}$.
$s^{\prime }$	Derived seed used for the re-optimization call at this emergency event (deterministic function of *s*, $t_{E}^{arr}$, and the emergency index).
MergeAndShift (·)	Procedure that merges frozen tasks from $S_{cur}^{det}$ with the new detailed schedule $S_{new}^{det}$ by shifting tasks forward to satisfy (i) start after $t_{E}^{arr}$, (ii) room availability induced by frozen tasks, and (iii) intra-job precedence.

**Table 5:** Elective surgical workload composition: clinical procedure types and nominal processing durations (min) for each operative stage.

Job Index (*j*)	Procedure *(Clinical Type)*	Processing Time (min)		
		Op. 1 *(Anesthesia)*	Op. 2 *(Surgery)*	Op. 3 *(Recovery)*
1	Laparoscopic Cholecystectomy	30	60	40
2	Laparoscopic Cholecystectomy	40	60	40
3	Laparoscopic Cholecystectomy	35	80	40
4	Common Bile Duct Expl.	65	190	60
5	Common Bile Duct Expl.	70	190	60
6	Common Bile Duct Expl.	75	190	60
7	Open Cholecystectomy	80	150	50
8	Open Cholecystectomy	70	110	50
9	Laparoscopic Cholecystectomy	35	80	40
10	Laparoscopic Cholecystectomy	30	80	40
11	Open Cholecystectomy	60	110	50
12	Open Cholecystectomy	80	110	50
13	Common Bile Duct Expl.	65	210	60
14	Laparoscopic Cholecystectomy	40	70	40
15	Common Bile Duct Expl.	70	160	60

**Table 6:** Simulation parameters and cost function coefficients.

Parameter	Symbol	Value
*Operational Constants*		
Setup Time	$t^{s}$	30 min
Cleanup Time	$t^{c}$	20 min
Max Emergency Delay	-	60 min
*Cost Function Coefficients*		
Makespan Weight	-	1.0
Start Time Weight	α	$10^{-6}$
Total Waiting Weight	β	0.7
Max Waiting Weight	γ	1.4
Imbalance Penalty	δ	100.0

**Table 7:** Parameter settings and search space configuration for the evaluated metaheuristics.

Algorithm	Parameter	Value
**Common**	Population/Swarm Size	30
	Max Iterations	1000
	Independent Replications	300
**GA**	Crossover Probability	0.8
	Mutation Probability	0.3
	Elitism Count	2
**dPSO**	Inertia Weight (*w*)	0.7
	Acceleration Coeffs ($c_{1},c_{2}$)	1.5
	Velocity Bounds	[−4,4]
**SBOA**	Search Domain	[−5,5]
**dMShOA**	Parameter *k*	0.3
	Search Domain	[−5,5]

**Table 8:** Summary of performance statistics (makespan in minutes and CPU time in seconds) across 300 replications, highlighting central tendencies and operational variability.

Algorithm	Min	Median	Mean (μ)	Std. Dev. (σ)	CV (%)	CPU Time (s)
GA	894.04	1162.70	1174.12	114.30	9.73%	12.93
dPSO	944.16	1179.52	1196.82	125.71	10.50%	15.36
SBOA	938.38	1175.84	1188.79	132.36	11.13%	23.99
dMShOA	938.24	1161.26	1180.21	127.65	10.81%	13.71

**Table 9:** Reference processing-time profiles for emergency duration parameterization (minutes). Durations are sampled around these baselines with a 10% standard deviation.

Procedure Type (Reference Job)	Op. 1 (Anesth.)	Op. 2 (Surg.)	Op. 3 (Recov.)
Laparoscopic Cholecystectomy (Type 1)	30	60	40
Open Cholecystectomy (Type 2)	80	150	50
Common Bile Duct Expl. (Type 3)	65	190	60

**Table 10:** Latency metrics in emergency integration (minutes). *Start* ($x_{E_{1}}^{r}$) denotes the simulator timestamp at which the emergency’s operation 1 begins its setup; $D_{int}=x_{E_{1}}^{r}-t_{E}^{arr}$.

Algorithm	Emergency E16			Emergency E17		
	Arrival	Start	Delay	Arrival	Start	Delay
GA	436.12	466.47	30.35	642.08	642.08	**0.00**
dPSO	552.60	552.60	**0.00**	596.06	675.92	79.86
SBOA	241.57	241.57	**0.00**	389.37	397.84	**8.47**
dMShOA	433.12	490.29	57.17	346.19	427.56	81.37

**Table 11:** Realized processing times (minutes) for the emergency cases in each algorithm’s best schedule. Values represent net processing ($p_{E,o}$).

Algorithm	E16: ($p_{E,1},p_{E,2},p_{E,3}$)			E17: ($p_{E,1},p_{E,2},p_{E,3}$)		
	Op.1	Op.2	Op.3	Op.1	Op.2	Op.3
GA	30.15	68.92	40.25	33.86	61.85	37.22
dPSO	65.91	146.01	54.26	34.72	58.17	35.36
SBOA	29.09	61.63	41.76	30.97	68.25	47.23
dMShOA	62.38	181.79	67.86	23.09	70.42	34.58

**Table 12:** Pairwise Mann-Whitney *U* test results for makespan distributions (n=300). Bold *p*-values indicate significance at α=0.05.

Comparison	U Statistic	*p*-Value	Effect Size (*r*)	Magnitude	Significant?
GA vs dPSO	40203.00	**0.0239**	0.11	Small	Yes
GA vs SBOA	42783.00	≥0.05	0.05	Trivial	No
GA vs dMShOA	44412.50	≥0.05	0.01	Trivial	No
dPSO vs SBOA	47225.50	≥0.05	-0.05	Trivial	No
dPSO vs dMShOA	49042.50	≥0.05	-0.09	Trivial	No
SBOA vs dMShOA	46655.50	≥0.05	-0.04	Trivial	No

**Table 13:** Summary of Best-Case Performance Metrics across 300 Replications.

Algorithm	Best $C_{\max}$ (min)	Avg. CPU (s)	Best Strategy Type
GA	894.04	12.93	Aggressive Compaction
dPSO	944.16	15.36	Dispersed Allocation
SBOA	938.38	23.99	Adaptive Flexibility
dMShOA	938.24	13.71	Balanced Exploitation

**Table 14:** GA Best Sequencing Strategy (Simulation #41).

Room	Operation Sequence (Job ID and Stage)
APR_1	3(Op1) → 14(Op1) → E17(Op1)
APR_2	15(Op1) → 12(Op1) → 4(Op1) → 2(Op1) → E16(Op1)
APR_3	13(Op1) → 6(Op1) → 8(Op1) → 5(Op1) → 11(Op1)
APR_4	1(Op1) → 10(Op1) → 9(Op1) → 7(Op1)
OR_1	1(Op2) → 6(Op2) → 9(Op2) → E16(Op2) → E17(Op2)
OR_2	13(Op2) → 8(Op2) → 2(Op2) → 11(Op2)
OR_3	15(Op2) → 10(Op2) → 4(Op2) → 7(Op2)
OR_4	3(Op2) → 12(Op2) → 5(Op2) → 14(Op2)
ARR_1	3(Op3) → 15(Op3) → 10(Op3) → 2(Op3) → 11(Op3) → E17(Op3)
ARR_2	6(Op3) → 9(Op3) → 5(Op3) → 7(Op3)
ARR_3	12(Op3) → 8(Op3) → 4(Op3) → E16(Op3)
ARR_4	1(Op3) → 13(Op3) → 14(Op3)

**Table 15:** Detailed surgical operation sequence and resource mapping for the dPSO optimal schedule (Simulation #208).

Room	Operation Sequence (Job ID and Stage)
APR_1	1(Op1) → 8(Op1) → E17(Op1)
APR_2	11(Op1) → E16(Op1)
APR_3	10(Op1) → 15(Op1) → 6(Op1) → 14(Op1) → 7(Op1) → 3(Op1)
APR_4	2(Op1) → 13(Op1) → 5(Op1) → 12(Op1) → 9(Op1) → 4(Op1)
OR_1	11(Op2) → 5(Op2) → 9(Op2) → 3(Op2) → E17(Op2)
OR_2	10(Op2) → 15(Op2) → 14(Op2) → 7(Op2) → 8(Op2)
OR_3	2(Op2) → 1(Op2) → 13(Op2) → 12(Op2) → 6(Op2) → E16(Op2)
OR_4	4(Op2)
ARR_1	10(Op3) → 3(Op3) → E17(Op3)
ARR_2	14(Op3) → 5(Op3) → 7(Op3) → 8(Op3)
ARR_3	2(Op3) → 1(Op3) → 12(Op3) → 9(Op3) → 4(Op3) → E16(Op3)
ARR_4	11(Op3) → 15(Op3) → 13(Op3) → 6(Op3)

**Table 16:** Detailed surgical operation sequence and resource mapping for the SBOA optimal schedule (Simulation #169).

Room	Operation Sequence (Job ID and Stage)
APR_1	4(Op1) → 8(Op1) → E16(Op1) → 2(Op1) → 7(Op1)
APR_2	15(Op1) → 14(Op1) → 5(Op1)
APR_3	9(Op1) → 1(Op1) → 11(Op1) → 12(Op1)
APR_4	6(Op1) → 13(Op1) → 10(Op1) → 3(Op1) → E17(Op1)
OR_1	6(Op2) → 15(Op2) → 2(Op2) → 7(Op2)
OR_2	4(Op2) → 10(Op2) → 3(Op2) → 11(Op2)
OR_3	9(Op2) → 13(Op2) → 1(Op2) → 5(Op2)
OR_4	8(Op2) → 14(Op2) → 12(Op2) → E16(Op2) → E17(Op2)
ARR_1	6(Op3) → 10(Op3) → 15(Op3) → E16(Op3) → 1(Op3) → 12(Op3)
ARR_2	8(Op3) → 14(Op3) → 7(Op3)
ARR_3	13(Op3) → 2(Op3) → E17(Op3) → 5(Op3)
ARR_4	9(Op3) → 4(Op3) → 3(Op3) → 11(Op3)

**Table 17:** Detailed surgical operation sequence and resource mapping for the dMShOA optimal schedule (Simulation #68).

Room	Operation Sequence (Job ID and Stage)
APR_1	1(Op1) → 13(Op1) → 10(Op1) → 2(Op1) → 15(Op1) → E16(Op1)
APR_2	3(Op1) → 7(Op1) → 4(Op1) → 6(Op1) → 5(Op1)
APR_3	9(Op1) → 12(Op1) → E17(Op1)
APR_4	11(Op1) → 14(Op1) → 8(Op1)
OR_1	11(Op2) → 10(Op2) → 12(Op2) → E16(Op2)
OR_2	1(Op2) → 13(Op2) → 2(Op2) → 8(Op2) → 5(Op2)
OR_3	9(Op2) → 14(Op2) → 4(Op2) → 15(Op2)
OR_4	3(Op2) → 7(Op2) → 6(Op2) → E17(Op2)
ARR_1	13(Op3) → E17(Op3) → 5(Op3)
ARR_2	8(Op3) → E16(Op3)
ARR_3	1(Op3) → 3(Op3) → 14(Op3) → 7(Op3) → 2(Op3) → 12(Op3) → 15(Op3)
ARR_4	9(Op3) → 11(Op3) → 10(Op3) → 4(Op3) → 6(Op3)

## Data Availability

The source code and the simulation framework developed in this study are openly available in the GitHub repository: https://github.com/imaberro/Surgical-Scheduling-GA-dPSO-SBOA-dMSHOA.git. This repository includes the implementation of the metaheuristics, the mathematical model configurations, and the scripts used to generate the results presented in this manuscript to ensure full reproducibility. Accessed on 13 January 2026.

## Abbreviations

The following abbreviations are used in this manuscript:

SS	Surgical Scheduling
JSSP	Job Shop Scheduling Problem
FJSSP	Flexible Job Shop Scheduling Problem
MIP	Mixed-Integer Programming
GA	Genetic Algorithm
PSO	Particle Swarm Optimization
dPSO	Discrete Particle Swarm Optimization
SBOA	Secretary Bird Optimization Algorithm
MShOA	Mantis Shrimp Optimization Algorithm
dMShOA	Discrete Mantis Shrimp Optimization Algorithm
APR	Anesthesia Preparation Room
OR	Operating Room
ARR	Post-Anesthesia Recovery Room
PACU	Post-Anesthesia Care Unit
ICU	Intensive Care Unit
LSTM	Long Short-Term Memory
OX	Order-based Crossover
IQR	Interquartile Range
CV	Coefficient of Variation
PRNG	Pseudo-Random Number Generator
GES	Explicit Health Guarantees (Garantías Explícitas en Salud)
COMGES	Management Commitments (Compromisos de Gestión)
